# Diffusion-Weighted Magnetic Resonance Imaging of Patients with Breast Cancer Following Neoadjuvant Chemotherapy Provides Early Prediction of Pathological Response – A Prospective Study

**DOI:** 10.1038/s41598-019-52785-3

**Published:** 2019-11-08

**Authors:** Nara P. Pereira, Carla Curi, Cynthia A. B. T. Osório, Elvira F. Marques, Fabiana B. Makdissi, Katja Pinker, Almir G. V. Bitencourt

**Affiliations:** 1 Department of Imaging – A.C.Camargo Cancer Center, R. Prof. Antônio Prudente, 211. Zip Code: 01509-010, São Paulo, SP Brazil; 2 Breast Cancer Reference Center – A.C.Camargo Cancer Center, R. Prof. Antônio Prudente, 211. Zip Code: 01509-010, São Paulo, SP Brazil; 3 Department of Pathology – A.C.Camargo Cancer Center, R. Prof. Antônio Prudente, 211. Zip Code: 01509-010, São Paulo, SP Brazil; 4 Department of Radiology, Breast Imaging Service – Memorial Sloan-Kettering Cancer Center 300 E 66th St. Zip Code, 10065 New York, NY USA

**Keywords:** Breast cancer, Cancer imaging

## Abstract

The purpose of this study was to evaluate the capacity of diffusion-weighted magnetic resonance imaging (DW-MRI) for early prediction of pathological response in breast cancer patients undergoing neoadjuvant chemotherapy (NCT). This prospective unicentric study evaluated 62 patients who underwent NCT. MRI was performed prior to the start of treatment (MR1), after the first NCT cycle (MR2), and upon completion of NCT (MR3). Pathological response was used as the gold-standard. Patients’ median age was 45.5 years and the median tumor size was 40 mm. Twenty-four (38.7%) tumors presented complete pathological response (pCR). The percent increase in apparent diffusion coefficient (ADC) value between MR1 and MR2 was higher in the pCR group (p < 0.001). When the minimum increase in ADC between MR1 and MR2 was set at 25%, sensitivity was 83%, specificity was 84%, positive predictive value was 77%, negative predictive value was 89%, and accuracy was 84% for an early prediction of pCR to NCT. Meanwhile, there were no significant changes in major tumor dimensions between MR1 and MR2. In conclusion, an increase in ADC after the first cycle of NCT correlates well with pCR after the chemotherapy in our cohort, precedes reduction in tumor size on conventional MRI, and may therefore be used as an early predictor of treatment response.

## Introduction

Neoadjuvant chemotherapy (NCT) is nowadays widely used for breast cancer treatment, even in women who are eligible for breast conservation at diagnosis. Early evaluation of response to this treatment could improve treatment efficiency and provide a more individualized management of the disease. Magnetic resonance imaging (MRI) is the best imaging method to evaluate response to NCT and predict pathologic response. However, conventional dynamic contrast-enhanced (DCE) MRI has a limited role in the early response assessment after the first cycles of NCT^1^.

Diffusion-weighted magnetic resonance imaging (DW-MRI) measures the mobility of water molecules in tissues, thereby providing information regarding the cellularity and integrity of cell membranes. DW-MRI has been shown to increase the diagnostic accuracy of DCE-MRI. In addition, it facilitates assessments of tumor response to NCT with a response being associated with a decrease in cellularity and an increase in apparent diffusion coefficient (ADC) values^2–4^.

DW-MRI allows tumor response to be evaluated *in vivo* in a non-invasive manner and it can improve individual treatments according to the degree of response observed. An evaluation of residual cancer after NCT is essential for determining a patient’s prognosis and appropriate clinical/surgical management^2^. Previously, DWI has been evaluated for its ability to provide an early prediction of tumor response in patients who undergo NCT^5^. However, direct comparisons among these studies to confirm the ability of DWI to reliably provide tumor predictions have been hindered by: methodological conflicts regarding technical parameters used, the variety of methods available for estimating tumor size, the use of multiple regions of interest in a tumor to quantify ADC and temporal change, and an insufficient number of cases studied. These limitations have also prevented widespread application of DWI for patients undergoing NCT for breast cancer^6–14^.

The aim of this study was to prospectively evaluate the use of DW-MRI for obtaining an early prediction of pathological response in patients with breast cancer before, during, and after NCT.

## Methods

### Patient selection

This prospective unicentric study was approved by Research ethics committee of the A.C.Camargo Cancer Center and all patients gave written, informed consent. 123 patients aged between 27 and 65 years (median 45 years) with biopsy-proven invasive breast cancer who underwent NCT between January 2015 and July 2016 were evaluated for inclusion in this study. The inclusion criteria for this study were: female patients with biopsy-proven breast cancer; ≥18 years; not pregnant; not breastfeeding; treatment with NCT with all treatments completed at the same institution; multiparametric MRI performedbefore, during (after 1^st^ cycle), and after completion of treatment;. Patients were excluded if they had undergone MRI or any treatment at another institution and if there were contraindications for MRI or MRI contrast agents.

Sixty-one patients (49%) were excluded because pretreatment MRI was not performed at our institution (n = 10), they received a diagnosis of distant metastasis (n = 4), they underwent chemotherapy follow-up at another institution (n = 8), they did not consent to participate (n = 5), or they did not show up for a scheduled exam (n = 34). Therefore, a total of 62 (51%) patients were included in this study (median age: 45.5 y; range: 27–65 y).

For all patients age, type and start date of systemic therapy,histologic type, tumor grade, receptor status; tumor proliferation rate (ki67), nodal status were recorded at commencement of NCT. All patients underwent NCT followed by surgery. The NCT regimen consisted of four cycles of anthracycline and cyclophosphamide at intervals of 3–4 weeks followed by four cycles of weekly paclitaxel (AC-T) (n = 37). Trastuzumab was added to this regimen (AC-T) in the16 cases with HER-2 overexpression. Other treatments were performed on a case-by-case basis, such as the addition of carboplatin to AC-T (n = 8) and the addition of pertuzumab to trastuzumab and docetaxel (n = 1).

### MR imaging

Each patient underwent MRI prior to NCT (MR1), after the first cycle of treatment and before the second cycle of treatment (MR2), and after completing NCT (MR3). Multiparametric breast MRI was performed in the prone position with a 1.5 T MR imaging system (GE Medical Systems, Milwaukee, WI, USA and Achieva, Philips Healthcare, Best, Netherlands) with a 8-channel dedicated breast coil. Patients were in a prone position to obtain images before and after administration of 20 ml of gadopentetate dimeglumine (Bayer Health Care Pharmaceuticals, Osaka, Japan) at an injection rate of 3 mL/s. DW images of both breasts were acquired in the transverse plane. A spin-echo, single-shot echo planar imaging sequence with diffusion-sensitizing gradients applied in the orthogonal directions was used. DW imaging also included two b-values, 0 s/mm^2^ and 750 s/mm^2^, with the latter recommended in a previous study^15^. After DWI, five three-dimensional (3D), T1-weighted gradient-echo sequences with fat suppression were obtained in the axial plane. The first image was obtained prior to the administration of contrast reagent and the last sequence consisted of post-contrast images.

### Image analysis

Three radiologists evaluated the MR images that were obtained at each of three exams (MR1–3). For early response assessment, only MR1 and MR2 images were evaluated. The radiologists were blinded to patient clinical information, tumor biological characteristics (such as histological grade and hormone receptor status), and NCT response.

#### DCE-MRI

For each patient, their primary tumor was initially identified in first-minute post-contrast fat-sat T1-weighted images with pre- and post-contrast subtraction. The longest diameter of the lesions was measured from the DCE-MR images and Response Evaluation Criteria in Solid Tumors (RECIST) guidelines were used to classify patients as responders or non-responders^16^.

#### DW-MRI

DW images and ADC maps were correlated with DCE-MR images. To detect residual disease on DW images after NCT, regions with signals greater than that of healthy breast parenchyma were considered to be positive. After identifying hypointense tumor regions in the ADC map, a two-dimensional region of interest (ROI) was manually drawn on the lesion avoiding normal fibroglandular parenchyma, adipose tissue, and necrotic areas.

ADC values were calculated according to the following equation and a linear regression model: ADC = [ln(S_0_/S_D_)]/*b*_1_ − *b*_0_), where b_1_ is the minimum value of b (0 sec/mm^2^), b_2_ is the maximum value of b (750 sec/mm^2^), and S_0_ and S_D_ are the signal strengths obtained for the ROI in different gradients in which the repetition time (TR) and the echo time (TE) remain constant. Mean ADC values for each ROI and for each tumor were calculated. Percent change in ADC (ΔADC%) was calculated as follows:

ADC% = [(ADC_post_ − ADC_pre_)/ADC_pre_] × 100, where ADC_pre_ and ADC_post_ represent pre- and post-treatment ADC values measured on MRI 1 and MR2, respectively. ADC values were analyzed by using an ADC map (Advantage Workstation, version 4.2, GE Healthcare).

### Histopathologic analysis

In all patients breast cancer was diagnosed by core needle biopsy. Histologic grade, estrogen receptor (ER) and progesterone receptor (PR) status, and human epidermal growth factor receptor (HER)−2 expression were obtained from histopathologic reports of core needle biopsies that were performed before chemotherapy. Tumors were classified based on immunohistochemistry results in one of the following molecular subtypes: Luminal A (ER-positive, Ki-67 < 20%, and HER-2-negative); Luminal B (ER-positive with either Ki-67 ≥ 20 or HER-2-positive); HER-2 enriched (ER-negative and HER-2-positive); and triple-negative (ER-negative, HER-2-negative, and PR-negative). The histologic findings of the 62 lesions prior to NCT were no special type (NST) invasive carcinomas (n = 53), invasive lobular carcinoma (n = 7), and other special types (n = 2). Thirty (48.4%) tumors were positive for ER expression, 28 (45.2%) tumors were positive for PR expression, and 17 tumors (27.4%) were HER-2-positive neoplasms. Regarding molecular subtypes, 35.5% (22/62) of the tumors were triple-negative, 16.1% (10/62) were HER-2 enriched, 37.1% (23/62) were luminal B–Ki-67, and 11.3% (7/62) were luminal B-HER2. For our statistical analyses, the latter two groups of tumors were combined in the same “Luminal B” group. A final pathologic examination was also conducted following the last cycle of chemotherapy after surgical excision was performed.

The majority of patients in our cohort (n = 35, 56.5%) underwent mastectomy, while 27 patients (43.5%) underwent conservative surgery. Pathologic responses were defined as complete pathological response (pCR) [with residual tumor completely absent in the breast and axilla (ypT0 and pN0), independent of the presence of ductal carcinoma *in situ*] and an absence of complete pathological response (non-pCR) [with residual invasive disease observed in the breast and axilla (ypT1-T4 and ypN1-N3)].

Pathological responses were evaluated according to the Residual Cancer Burden (RCB) protocol^17^. Briefly, for each surgical specimen, four variables were evaluated: the primary dimension of the tumor bed, the cellularity fraction of the invasive component, the dimension of the largest metastasis, and the number of axillary lymph nodes involved. These data were entered into the RCB calculator of the MD Anderson Cancer Center website (https://www.mdanderson.org/for-physicians/clinical-tools-resources/clinical-calculators/residual-cancer-burden.html) which automatically calculates a value as follows: (a) RCB-0 = pCR; (b) RCB-I = minimal residual disease; (c) RCB-II = moderate residual disease; or (d) RCB-III = extensive residual disease.

### Statistical analysis

Response rates obtained from DWI/DCE MR imaging and pathological response of surgical specimens were correlated and evaluated with SPSS for Windows (version 20.0). Sensitivity, specificity, positive predictive value (PPV), negative predictive value (NPV), and accuracy of DCE and DWI for tumor dimensions were calculated. ADC change at MR1-2 was examined, with pathological response used as a gold standard. A receiver operating characteristic (ROC) curve was used to determine the cutoff value for ADC change that best classified the tumors as pCR or non-pCR. The best cutoff value was defined as that which provided the highest average sensitivity and specificity values. *P*-values less than 0.05 were considered to indicate significant differences. The non-parametric Mann-Whitney test was used to analyze changes in ADC values and tumor size between two groups, while the non-parametric Kruskal-Wallis test was used to assess changes in ADC values and tumor size among three or more groups. The Spearman correlation coefficient (r) was used to correlate absolute ADC values with histopathological response values.

### Ethical considerations

The study was approved by the institutional ethics committee and all methods were carried out in accordance with relevant guidelines and regulations. Informed consent was obtained from all patients participating in the study.

## Results

### Pretreatment ADC Values

Table 1 lists the histopathological characteristics and pretreatment ADC values for the cases examined. After NCT, 24 tumors (38.7%) presented pCR. These tumors included triple-negative tumors (58%), HER-2 tumors (25%), and luminal B tumors (16.7%). The mean pretreatment ADC values did not significantly differ among the triple-negative (0.917 × 10^−3^ mm^2^/s), HER-2 overexpressing (0.834 × 10^−3^ mm^2^/s), and luminal B (0.795 × 10^−3^ mm^2^/s) tumors (p = 0.122). However, a statistically significant difference was observed between ADC values of the luminal B and triple-negative tumors (p = 0.003). There was no statistically significant difference between the pre-treatment ADC values in relation to histological grade and molecular subtypes. However, expression of progesterone and estrogen receptors did contribute to a lower ADC in MR1 (p = 0.02), while expression of HER-2 had no effect on ADC. There was also no statistically significant difference between the mean pretreatment ADC values of the pCR (0.832 ± 0.198 × 10^−3^ mm^2^/s) and non-pCR (0.853 ± 0.171 × 10^−3^ mm^2^/s) groups (p = 0.882).

**Table 1:** Histological characteristics and ADC values evaluated prior to NCT.

Histological Results	ADC MR1 average (×10^−3^ mm^2^/s)				
	Overall mean ADC (95% CI)	*Sig* ^1^	pCR (n = 24)	non-pCR (n = 38)	*Sig* ^2^
*Tumor histology*		*P* = 0.15			
Invasive ductal carcinoma	0.849 (0.483–1.330)		0.854 (0.596–1.330)	0.845 (0.483–1.280)	*P* = 0.601
Invasive lobular carcinoma	0.749 (0.448–0.988)		0.593 (0.448–0.738)	0.811(0.699–0.988)	*P* = 0.190
Others	1.073 (0.756–1.390)		—	1.07 (0.756–1.390)	—
*Histologic grade (SBC)*		*P* = 0.10			
II	0.884 (0.483–1.390)		0.925 (0.751–1.170)	0.870 (0.483–1.39)	*P* = 0.518
III	0.809 (0.448–1.330)		0.786 (0.448–1.330)	0.831 (0.620–1.030)	*P* = 0.191
*Estrogen receptor*		*P* = 0.02			
Positive	0.789 (0.483–1.090)		0.751 (0.596–0.103)	0.796 (0.483–1.090)	*P* = 0.275
Negative	0.897 (0.448–1.390)		0.853 (0.448–1.330)	0.961 (0.739–1.39)	*P* = 0.071
Progesterone receptor		*P* = 0.02			
Positive	0.782 (0.483–1.090)		0.663 (0.596–0.751)	0.796 (0.483–1.090)	*P* = 0.051
Negative	0.896 (0.448–1.390)		0.857 (0.448–1.330)	0.961 (0.739–1.39)	*P* = 0.082
*HER-2*		P = 0.75			
Positive	0.823 (0.651–1.140)		0.831 (0.655–0.988)	0.826 (0.651–1.140)	*P* = 0.733
Negative	0.851 (0.448–1.390)		0.862 (0.483–1.390)	0.834 (0.448–1.330)	*P* = 0.451
*Tumor phenotype*		*P* = 0.12			
Luminal B	0.795 (0.483–1.090)		0.755 (0.596–1.035)	0.802 (0.483–1.090)	*P* = 0.359
Triple-negative	0.917 (0.448–1.390)		0.857 (0.448–1.330)	1.02 (0.739–1.390)	*P* = 0.070
HER-2	0.834 (0.651–1.140)		0.826 (0.651–1.140)	0.847 (0.772–0.949)	*P* = 0.522

Note: Sig^1^ refers to statistical significance between the categories in the left column; Sig^2^ refers to statistical significance between the pCR and non-pCR groups in the same line.

Statistical significance determined with Mann-Whitney test, P = 0.05.

ADC: apparent diffusion coefficient; NCT: neoadjuvant chemotherapy; MR1: MR exam prior to treatment; CI: confidence interval; pCR: complete pathological response; non-pCR: an absence of complete pathological response.

### Evaluation of ADC for treatment response

**Figure 1:**
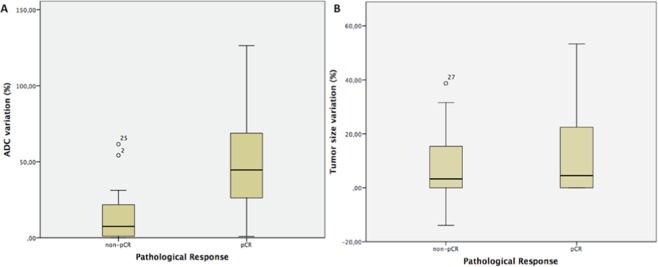
(**A**,**B**) Box and whisker plots of changes in (**A**) ADC values and (**B**) tumor size before and after the first cycle of treatment (MR1 vs. MR2). The horizontal line within each box represents the median value. In (**A**), the outliers represent two non-responder cases: 1) ΔADC = 54.24%, there was a complete response in the breast lesion, however a positive axillary lymph node remained after surgery, thereby resulting in a non-pCR classification; 2) ΔADC = 61.56%, a substantial inflammatory effect was observed after the first cycle of NCT, and this resulted in high T2 signal intensity, an increase in ADC value, and an increase in breast enhancement, despite the absence of a complete pathological response after treatment. The morphology of both cases indicated non-mass enhancement lesions. In (**B**), the outlier represents a non-mass enhancement tumor that was fragmented and showed scattered areas throughout the tumor burden in MR2.

Table 2 lists the mean ADC values for MR1 and MR2. The mean ADC value for the pCR group at MR2 was significantly higher than the mean ADC value at MR1 (p < 0.001). Table 2 also shows the ΔADC% increase and ΔSize% decrease between MR1 and MR2. In Fig. 1, box plot graphs of ADC values and size changes between MR1 and MR2 are presented. The mean ΔADC% increase for the pCR group was significantly greater than that for the non-pCR group (p < 0.001).

**Table 2:** ΔADC determined from ADC values at MR1 and MR2 versus ΔSize are presented as mean ± standard error.

Pathological response	ADC MR 1 (× 10^–3^)	ADC MR 2 (× 10^−3^)	ΔADC (%)	ΔSize (%)
pCR	0.832 ± 0.044	1.214 ± 0.0599	44.36 ± 6.7	4.5 ± 3.3
Non-pCR	0.853 ± 0.027	0.954 ± 0.0267	7.54 ± 2.3	3.3 ± 1.7
*Sig* ^*^	P = 0.882	**P** < **0.001**	**P** < **0.001**	P = 0.457

^*^According to Mann-Whitney test

ADC: apparent diffusion coefficient; NCT: neoadjuvant chemotherapy; MR1: MR exam prior to treatment; MR2: MR exam after first cycle of treatment; pCR: complete pathological response; non-pCR: an absence of complete pathological response.

There was a statistically significant difference in ΔADC% between MR1 and MR2 for the triple-negative and luminal B cases. The triple-negative cases also showed a significant increase in ADC values between MR1 and MR3. In contrast, the ADC values of the HER-2 subtype cases did not exhibit a statistically significant difference in any of the exams, while the luminal B cases only showed a significant change between MR1 and MR2 (Table 3).

**Table 3:** Percentage increase in ADC (∆ADC) and percentage decrease in tumor size (∆Size) after the first cycle of neoadjuvant chemotherapy for pCR and non-pCR groups, according to tumor subgroup.

Subgroup	MRI	pCR	non-pCR	*Sig*
*Triple-negative*	∆ADC	53%	7%	P = 0.002
	∆Size^*^	16%	6%	P = 0.27
*Luminal B*	∆ADC	42%	16%	P = 0.009
	∆Size^*^	12%	8%	P = 0.47
*HER2 overexpression*	∆ADC	43%	7%	P = 0.055
	∆Size^*^	7%	7.5%	P = 1.00

^*^Measurements obtained from DCE-MRI.

ADC: apparent diffusion coefficient; NCT: neoadjuvant chemotherapy; MR1: prior to first cycle of treatment; MR2: after first cycle of treatment; MR3: after final cycle of treatment; pCR: complete pathological response; non-pCR: an absence of complete pathological response. Sig: According to the Kruskal-Wallis test.

**Figure 2:**
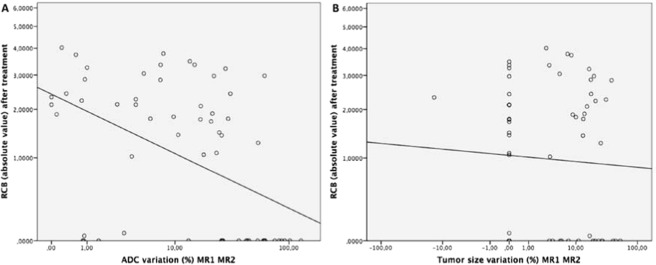
(**A**,**B**) Scatter plots show correlations between absolute RCB values after treatment and ADC (**A**) and changes in tumor size (**B**) between MR 1 and MR2 for 62 patients. The trend lines in each panel represent the least-squares fit for the data. (**A**) A moderate inverse correlation is observed between the RCB and ADC values (*ρ* = −0.553). (**B**) A weak correlation is observed between the RCB and size change values (*ρ* = −0.052). There were many lesions that were stable in size that had multiple RCB values.

Scatter plots of ΔADC% in MR1 and MR2 and absolute RCB values did show a moderate correlation between these data (r = 0.553; p < 0.001) (Fig. 2).

**Figure 3:**
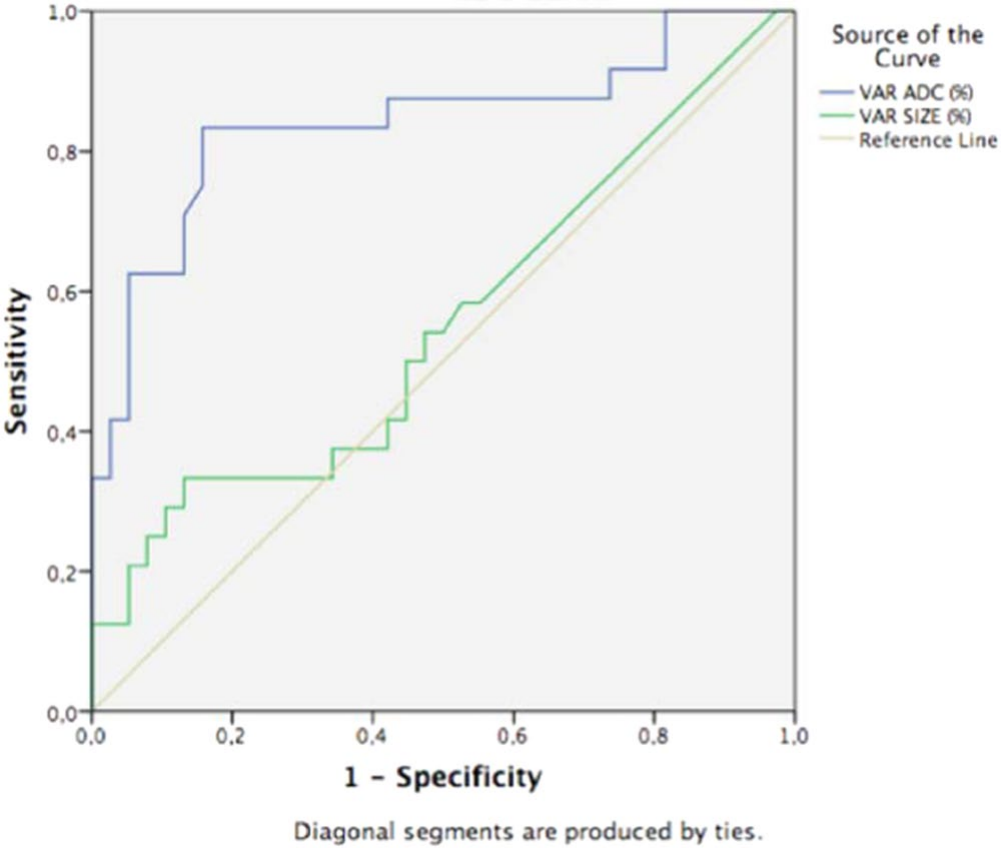
ROC curves for using ADC (blue line) or DCE (green line) as predictors of early response in 62 patients with invasive breast cancer. The best percent ADC increase cutoff value for differentiating responders from non-responders after the first cycle of NCT was 25%, and the area under the ROC curve was 0.840 (95% confidence interval: 0.728, 0.953). The latter had a greater area under the curve compared with tumor size, thereby indicating that DW-MRI is a more effective method for predicting early NCT response than evaluating tumor size with DCE-MRI.

A ROC curve analysis showed that the best ΔADC% cutoff value for predicting pCR was 25%, and the area under the ROC curve was 0.840 (95% confidence interval: 0.728, p = 0.953). This cutoff value yielded a sensitivity value of 83%, a specificity value of 84%, a PPV of 77%, and a NPV of 89% for the use of DW-MRI. In addition, six false-positive cases were identified with this cutoff value (Fig. 3). Considering only the NST invasive carcinomas (n = 52), the results were similar with an area under the ROC curve of 0.822 and a sensitivity and specificity of 81.8% and 80.6%, respectively, using the same ΔADC% cutoff value.

### DCE-MRI analysis

**Figure 4:**
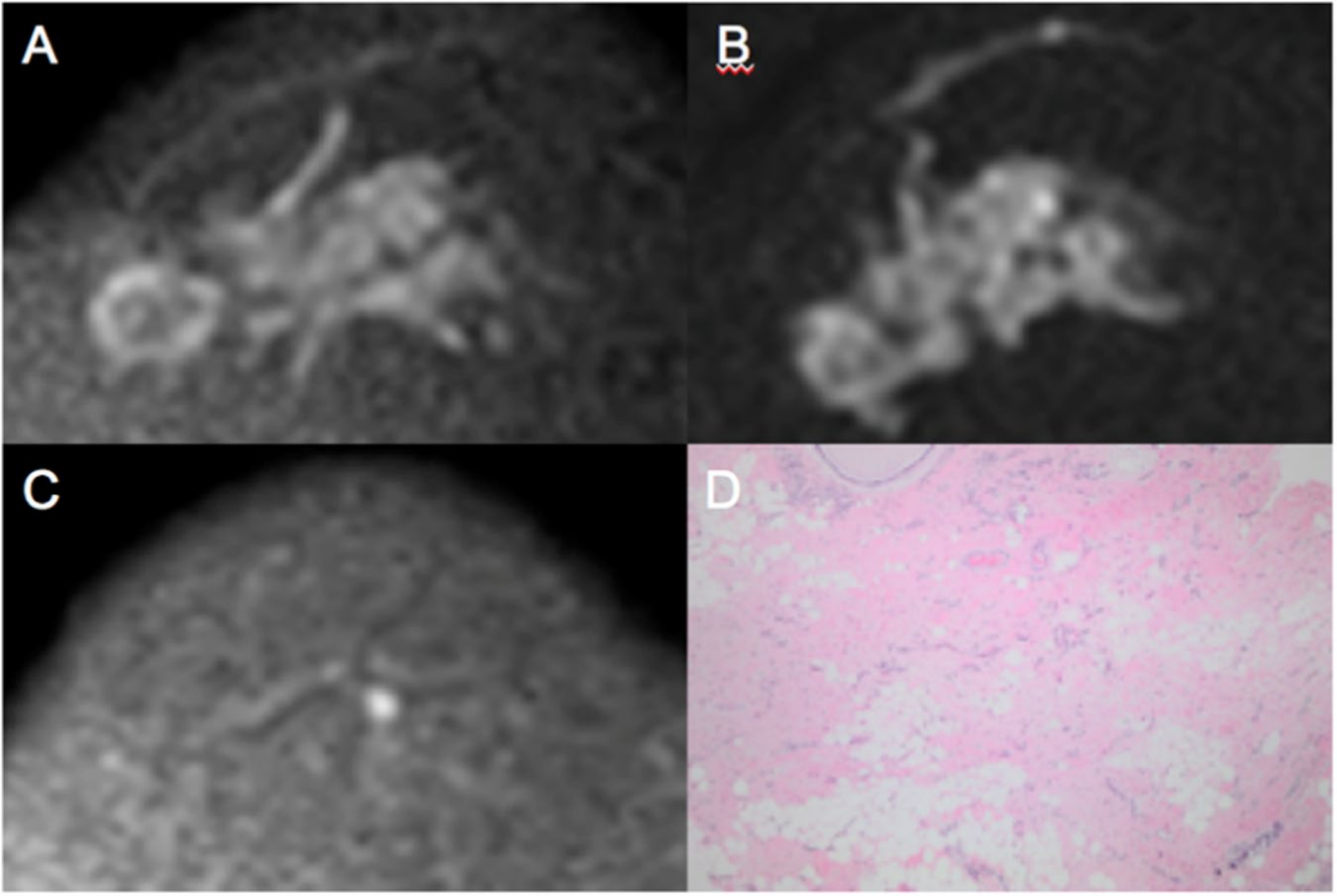
Images obtained from a 58-year-old female diagnosed with LABC HER-2 breast cancer who presented pCR after mastectomy. The ΔADC% increase between MR1 and MR2 was 54% (true positive to early prediction of NCT). No size change (false negative at this point) was observed after the first cycle of treatment. Because of its high restriction signal in diffusion, the patient was diagnosed with partial radiological response by DCE and DWI at MR3 (both false negative at this point). (**A**) Axial DWI at MR1 shows an extensive area with high signal intensity, denoting diffusion restriction. (**B**) Axial DWI at MR2 shows high signal intensity at the same site as shown in (**A**). (**C**) Axial DWI at MR3 shows a significant reduction in the area of diffusion restriction and high signal intensity at the tumor site. (**D**) Hematoxylin and eosin staining of a histological section of the mastectomy product after NCT. Residual disease is absent, while stromal fibrosis and vascular neoformation are observed, compatible with response to treatment (10x magnification).

Tumor size on pretreatment MR varied between 15 and 92 mm (median 38 mm). There was no statistically significant difference in the changes in tumor dimensions between MR1 and MR2 (Fig. 1 and Table 2; p = 0.457), and there was no statistically significant correlation between the difference in tumor size from MR1 to MR2 and absolute RCB values (Fig. 2 and Table 3; r = −0.052; p = 0.690). ROC curve analysis also showed that DCE-MRI did not detect a statistically significant decrease in tumor size to predict pCR after the first cycle of treatment (Mann-Whitney test; *p* = 0.474). The area under this ROC curve was 0.554 (95% confidence interval: 0.402, p = 0.706) (Fig. 3). Figure 4 presents a true positive case of an early prediction of pCR according to DWI, which was considered false-negative according to DCE images obtained at MR1 and MR2.

## Discussion

We investigated the application of DW-MRI for early prediction of pathological response in breast cancer patients after the first NCT cycle. We observed that the change in ADC, a functional parameter that provides information on intratumoral cellularity, between MR1 and MR2 was greater among the tumors that responded completely to treatment. Moreover, when the increase in ADC value was at least 25% between the first two MR exams. DW-MRI had a sensitivity of 83% and a specificity of 84% for an early prediction of pCR after NCT.

It is important to obtain feasible parameters to monitor NCT in breast cancer patients. DW-MRI and its functional parameter, ADC, are highly sensitive to changes in the cellular microenvironment in response to cytotoxic drugs. Moreover, a key advantage of DW-MRI is that it is a non-invasive method, it does not require contrast or ionizing radiation, image acquisition is achieved within a relatively short period of time, and it can be easily incorporated into routine clinical evaluations. The use of multiple drugs and the genetic variability of tumors remain major challenges in the standardization of ADC as a predictor of tumor response. However, the results of the present study demonstrate that DW-MRI represents a promising method for this purpose, especially when it is performed after the first cycle of chemotherapy. As a result, greater individualization of patient treatments can be achieved earlier, and this can potentially lead to higher rates of complete response at the end of therapy.

Some studies have evaluated ADC values from DW-MRI after one cycle of NCT^6,13,14,18,19^, and all of them found that the changes in ADC between the first two exams were good predictors of an early response to NCT for breast cancer. However, the results were not always statistically significant, probably due to the small number of cases that were evaluated in each study (56, 10, 24, 14, 11, 9 and 32 cases, respectively). Iwasa *et al*. were the first to report that an increase in ADC precedes a reduction in tumor size in NCT by comparing DW-MRI with ultrasound findings and correlating these results with complete response to therapy^6^. Jensen *et al*.^18^ also reported a significant increase in ADC values after a first cycle of treatment. In the latter study, the MR imaging parameters, DCE and ADC, were found to be altered earlier than changes in tumor size that were measured by physical examination. However, only ADC had a statistically significant result^18^.

Previously, in a meta-analysis that evaluated 15 studies which used MRI to assess tumors before and after four cycles of NCT, DWI was found to be superior to DCE in predicting complete response^5^. Moreover, in a study by Hu *et al*.^20^, both ADC and tumor size values were found to differ in the pCR group after a second cycle of NCT. In the present study, significant changes in ADC values were detected, while no significant change in tumor dimension was observed from the DCE images that were collected in parallel. These results confirm that functional variances in tumors precede morphological alteration, since we only detected the ADC alteration shortly after the first cycle and not the change in its maximum diameter.

Park *et al*. reported that tumors with lower ADC values prior to treatment respond better to NCT^21^. Correspondingly, in a study conducted by Richard *et al*., triple-negative tumors in a non-responders group exhibited significantly higher pretreatment ADC values^22^. In the present study, ADC values prior to treatment did not predict pathological response, and this is in agreement with other studies^23,24^. Moreover, when other prognostic pretreatment factors were examined, including tumor size, histological type, and degree of malignancy, the ADC values were not statistically significant for the purpose of predicting tumor biology, nor did they contribute to treatment selection or prognosis of response to NCT. These findings are also in agreement with other studies^25,26^.

There were limitations associated with the present study. First, specific conclusions regarding the biological effects of each treatment drug could not be evaluated based on changes in ADC value due to the heterogeneity of the chemotherapy treatments that were administered in our cohort. Second, there were fewer patients to examine due to difficulties in scheduling a second exam so early after the first cycle of chemotherapy and the small sample size could affect the results. Third, the Symmans protocol was used to evaluate pathological response to NCT, and ductal carcinoma *in situ* could be present in cases of pCR^17^. Thus, disagreements could arise between imaging and pathology. However, there were only two cases that achieved pCR that were associated with the presence of ductal carcinoma *in situ*. Finally, intratumoral ROIs were defined as regions most likely to contain a tumor, and this reduced the possibility of underestimating the ADCs of the lesions by discarding cystic areas and regions of necrosis.

In conclusion, a significant increase in ADC after the first cycle of NCT correlates well with pCR after the chemotherapy in our cohort of breast cancer patients, precedes reduction in tumor size on conventional DCE-MRI, and may therefore be used as an early predictor of treatment response.

## References

[1] Rauch GM (2017). Multimodality Imaging for Evaluating Response to Neoadjuvant Chemotherapy in Breast Cancer. AJR Am J Roentgenol..

[2] Chen JH, Su MY (2013). Clinical application of magnetic resonance imaging in management of breast cancer patients receiving neoadjuvant chemotherapy. BioMed Res Int..

[3] Lehman CD (2012). Diffusion weighted imaging (DWI) of the breast: ready for clinical practice?. Eur J Radiol..

[4] Kumar S, Badhe BA, Krishnan KM, Sagili H (2014). Study of tumour cellularity in locally advanced breast carcinoma on neo-adjuvant chemotherapy. J Clin Diagn Res JCDR..

[5] Chu W (2017). Diffusion-weighted imaging in identifying breast cancer pathological response to neoadjuvant chemotherapy: A meta-analysis. Oncotarget..

[6] Iwasa H, Kubota K, Hamada N, Nogami M, Nishioka A (2014). Early prediction of response to neoadjuvant chemotherapy in patients with breast cancer using diffusion-weighted imaging and gray-scale ultrasonography. Oncol Rep..

[7] Fujimoto H (2014). Diffusion-weighted imaging reflects pathological therapeutic response and relapse in breast cancer. Breast Cancer..

[8] Shin HJ (2012). Prediction of pathologic response to neoadjuvant chemotherapy in patients with breast cancer using diffusion-weighted imaging and MRS. NMR Biomed..

[9] Atuegwu NC (2011). Integration of diffusion-weighted MRI data and a simple mathematical model to predict breast tumor cellularity during neoadjuvant chemotherapy. Magn Reson Med..

[10] Belli P (2011). Diffusion-weighted imaging in evaluating the response to neoadjuvant breast cancer treatment. Breast J..

[11] Park SH (2012). Comparison of diffusion-weighted MR imaging and FDG PET/CT to predict pathological complete response to neoadjuvant chemotherapy in patients with breast cancer. Eur Radiol..

[12] Murata Y (2010). Diffusion-weighted magnetic resonance imaging for assessment after neoadjuvant chemotherapy in breast cancer, based on morphological concepts. Oncol Lett..

[13] Sharma U, Danishad KKA, Seenu V, Jagannathan NR (2009). Longitudinal study of the assessment by MRI and diffusion-weighted imaging of tumor response in patients with locally advanced breast cancer undergoing neoadjuvant chemotherapy. NMR Biomed..

[14] Pickles MD, Gibbs P, Lowry M, Turnbull LW (2006). Diffusion changes precede size reduction in neoadjuvant treatment of breast cancer. Magn Reson Imaging..

[15] Pereira FP (2009). Assessment of breast lesions with diffusion-weighted MRI: comparing the use of different b values. AJR Am J Roentgenol..

[16] Eisenhauer EA (2009). New response evaluation criteria in solid tumours: revised RECIST guideline (version 1.1). Eur J Cancer..

[17] Symmans WF (2007). Measurement of residual breast cancer burden to predict survival after neoadjuvant chemotherapy. J Clin Oncol..

[18] Jensen LR (2011). Diffusion-weighted and dynamic contrast-enhanced MRI in evaluation of early treatment effects during neoadjuvant chemotherapy in breast cancer patients. J Magn Reson imaging JMRI..

[19] Wilmes LJ (2013). High-resolution diffusion-weighted imaging for monitoring breast cancer treatment response. Acad Radiol..

[20] Hu X-Y (2017). Diffusion-weighted MR imaging in prediction of response to neoadjuvant chemotherapy in patients with breast cancer. Oncotarget..

[21] Park SH (2010). Diffusion-weighted MR imaging: pretreatment prediction of response to neoadjuvant chemotherapy in patients with breast cancer. Radiology..

[22] Richard R (2013). Diffusion-weighted MRI in pretreatment prediction of response to neoadjuvant chemotherapy in patients with breast cancer. Eur Radiol..

[23] Fangberget A (2011). Neoadjuvant chemotherapy in breast cancer-response evaluation and prediction of response to treatment using dynamic contrast-enhanced and diffusion-weighted MR imaging. Eur Radiol..

[24] Woodhams R (2010). Identification of residual breast carcinoma following neoadjuvant chemotherapy: diffusion-weighted imaging-comparison with contrast-enhanced MR imaging and pathologic findings. Radiology..

[25] Durando M (2016). Quantitative apparent diffusion coefficient measurement obtained by 3.0 Tesla MRI as a potential noninvasive marker of tumor aggressiveness in breast cancer. Eur J Radiol..

[26] Kim SH (2009). Diffusion-weighted imaging of breast cancer: correlation of the apparent diffusion coefficient value with prognostic factors. J Magn Reson Imaging JMRI..

